# DL_FFLUX Refactored: Accelerating Quantum Chemical Topology Simulations With Optimized Parallelization

**DOI:** 10.1002/jcc.70501

**Published:** 2026-09-08

**Authors:** Mohamadhosein Nosratjoo, Michael K. Bane, Paul L. A. Popelier

**Affiliations:** ^1^ Department of Chemistry The University of Manchester Manchester UK; ^2^ High End Compute LTD Manchester UK; ^3^ Department of Computing and Mathematics Manchester Metropolitan University Manchester UK

## Abstract

Molecular dynamics simulations are only as useful as they are both accurate and efficient. Here, we introduce an upgraded version of the simulation code DL_FFLUX that dramatically extends accessible time scales by combining a more efficient core algorithm with OpenMP and MPI parallelization. DL_FFLUX is a unique, state‐of‐the‐art polarisable machine learning potential (MLP) that combines Quantum Chemical Topology (QCT) and machine learning (ML) and relies on DL_POLY as the driver for molecular dynamics simulations. This new implementation of DL_FFLUX delivers a massive performance boost, making simulations 6.8 to 49 times faster without compromising accuracy. This performance boost of DL_FFLUX is reported for condensed‐phase simulations of water and ethanol and for single‐molecule simulations of 6 small to medium‐sized molecules. Moreover, we compared the optimized version of DL_FFLUX with other MLPs on the MD17 dataset for four molecules. DL_FFLUX is the fastest: depending on the system, it outperforms the second‐fastest MLP by a factor of 1.8–9.6.

## Graphical abstract

DL_FFLUX, which houses the quantum‐topological machine‐learning potential FFLUX, is refactored and computationally optimized through software‐level code optimization and prediction caching. The new implementation accelerates molecular dynamics simulations 93‐fold for the largest models while preserving accuracy. DL_FFLUX benchmarks as fastest among state‐of‐the‐art machine learning potentials for representative MD17 molecular systems.

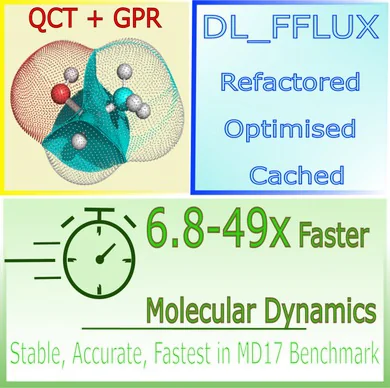

## 1 Introduction

For more than 70 years molecular modeling methods such as molecular dynamics (MD) [1, 2] and Monte Carlo (MC) [3] have been used extensively to study molecular systems in different areas such as chemistry, physics, material science or drug discovery [4, 5, 6, 7, 8]. These methods continue to be popular because of their computational efficiency, a trait that was vital during their early use when computational resources were limited. Nowadays, simulating hundreds of thousands or even millions of particles using various force fields in MD is routine. These force fields aim to model the potential energy surface (PES) of a system by decomposing it into two‐body terms for bonds, three‐body terms for angles, and four‐body terms for dihedral angles, and then using a set of limited empirical parameters within that potential energy function. However, capturing the complexity and quantum mechanical nature of the PES is often not possible with these simple potential energy functions. Moreover, the parameters are typically fitted to experimental or ab initio data sets to reproduce a limited range of specific thermodynamic properties. As a result, these force fields are limited in transferability and cannot accurately reproduce multiple properties [9].

In contrast, ab initio molecular dynamics (AIMD) [10, 11] leverages first‐principles electronic structure calculations, such as density functional theory (DFT), to compute energies and forces on the fly. At every MD step, an electronic‐structure calculation is performed to obtain the energy and forces, which are then used to propagate the nuclei; these calculations are therefore computationally expensive. While AIMD offers significantly higher accuracy, it suffers from high computational cost, limiting its applicability to smaller systems and short simulation timescales. This trade‐off between speed and accuracy has driven the search for more efficient approaches that retain the accuracy of AIMD while extending the accessibility of simulations to larger and more complex systems for longer timescales. Machine learning potentials (MLPs) have emerged as a powerful alternative, bridging the gap between classical MD/MC simulations and AIMD. In simple terms, MLPs learn the mapping between a set of features and an output. The gain in popularity of MLPs is because they are accurate, but also much faster than AIMD. Over the years, different groups have developed many MLPs [12, 13, 14, 15]. These MLPs differ in featurization, where some use handcrafted descriptors [16] and some have a pre‐training phase where the ML architecture generates a set of descriptors tailored to the training data [17]. Some use local descriptors, and some use global ones. The other aspect in which they are different is their ML architecture, such as neural networks [16, 17, 18, 19, 20, 21], permutationally invariant polynomials [22], and Gaussian process regression (GPR) [14].

Currently, most MLPs prioritize accuracy, frequently at the expense of computational speed. Achieving an accuracy of around 1 kcal/mol, commonly called “chemical accuracy,” has become increasingly routine. However, speed is the key advantage of MLPs over AIMD, and sacrificing it for more complex architectures can make them impractical for nanosecond to microsecond simulations. Additionally, MLPs remain substantially more costly to run than classical force fields [23].

Our in‐house MLP named FFLUX [24] is seamlessly interfaced with DL_POLY 4 [25] to form DL_FFLUX, a program capable of performing geometry optimization [26] and MD simulations of different systems [27, 28, 29] with ab initio accuracy at a fraction of the cost of the electronic calculations. FFLUX uses GPR [30] to predict not only the atomic energy but also atomic multipole moments up to the hexadecapole moment for geometries encountered in different steps of MD simulations. The atomic local frame (ALF) featurization, which is a global descriptor, is used to convert Cartesian coordinates into 3 N—6 features, where *N* is the number of atoms. The ALF ensures the invariance under transition and rotation, and more on ALF can be found elsewhere [31]. Here we show that by refactoring and optimizing the key parts of DL_FFLUX we have reversed this trend, without sacrificing accuracy, and made DL_FFLUX much faster for single molecules and condensed phase simulations.

## 2 Background

A number of preparative steps are necessary before carrying out MD simulations with FFLUX. Some of these steps are similar to other MLPs, and some are unique to FFLUX. The steps can be divided into separate categories of sampling, wavefunction generation, the calculation of atomic properties and training, which will be discussed in the following subsections.

### 2.1 Sampling

Sampling is the first step in any MLP, and arguably one of the most overlooked phases. MLPs learn the PES by training on datasets where the structures are usually generated using one normal mode or a combination thereof [32] or AIMD [33], classical MD [27, 34]. However, the initially sampled structures might be incomplete, which leads to holes in the PES [35]. This lack of coverage of conformational space is more pronounced in larger, more flexible systems. To address this issue, active learning schemes have been developed that were successful to some extent. Usually, in an active learning procedure, simulations are run using an initial model, and when an unphysical geometry is observed, the simulation is stopped. Those geometries are then added to the training dataset and a new model is generated. This process is performed iteratively until satisfactory stability and accuracy are achieved [36, 37]. The obvious drawback of this method is that the number of iterations required to achieve a satisfactory threshold is not known in advance. Moreover, the active‐learned data generated for a specific MLP may not perform as well when used with a different MLP [38]. It has also been observed that if the initial model is built with data that poorly sample the conformations, then the active learning procedure can become vastly more challenging [39]. These issues encourage research groups to use a complete dataset or one that is as complete as possible. This is why more recently, an increasing number of groups are using enhanced sampling techniques such as metadynamics [40, 41] or umbrella sampling [42] as their primary source of structure generation [43]. Metadynamics promotes uniform exploration of carefully chosen collective variables, typically the system's slowest motions, such as backbone dihedral angles, which reduces extrapolation in simulations and can eliminate it entirely during room temperature simulations.

After generating many geometries, it is common practice to perform subsampling to reduce the number of training points. This can be done in simple ways, such as random sampling, or by using more sophisticated methods, namely, procedures that maximize the diversity of the selected points [26].

### 2.2 Wavefunction Generation and Atomic Properties Calculation

The next phase after obtaining diverse geometries is the electronic calculations, which are done for them to form the dataset. In the case of FFLUX's training pipeline, after performing the electronic calculations and obtaining the wavefunction of the system, atomic energies, charges, and multipole moments are extracted from AIMAll [44], which implements several elements of the Quantum Theory of Atoms in Molecules (QTAIM) [45, 46].

At the core of FFLUX lies QTAIM and Interacting Quantum Atoms (IQA) [47], both of which are essential parts of the broader Quantum Chemical Topology (QCT) [48] framework. This framework encompasses various methods that utilize gradient vector field partitioning to analyze quantum mechanical functions. Partitioning the real‐space electron density yields quantum atoms, each associated with a well‐defined atomic basin. A 3D integration over the volume of a given basin of the electron density times the appropriate polynomial (e.g., xy) leads to the atomic multipole moments (MMs). FFLUX learns these atomic MMs and can predict them for geometries encountered in MD simulations. These MMs are termed flexible MMs since they change depending on the different geometries of the system at different steps of MD simulations.

One other feature that sets FFLUX apart from other MLPs is that, thanks to IQA, the energy is partitioned before training, which inherently embeds the physics into the data itself, providing an advantage for FFLUX. Within the IQA energy‐partitioning framework, an atom's total energy can be quantified even when the system is not at a stationary point on the PES. An atom's total energy is made up of its own intra‐atomic energy and the energy arising from interatomic interactions with other atoms. Equation (1) shows how molecular energy is broken into atomic IQA energies:

(1) $$E_{\mathrm{IQA}}^{\text{molec}}=\sum_{A}E_{\mathrm{IQA}}^{AA^{\prime }}=E_{\text{intra}}^{A}+\frac{1}{2}\sum_{B\neq A}^{N_{\text{atoms}}}E_{\text{inter}}^{\mathrm{AB}}$$

For atom *A*, the IQA energy $E_{\mathrm{IQA}}^{AA^{\prime }}$ uses the superscript $AA^{\prime }$ to show that it is made up of the intra‐atomic energy $E_{\text{intra}}^{A}$ plus the interaction energies between atom *A* and the other atoms $A^{\prime }$. Further breakdown of intra‐ and interatomic terms is possible [49]. In FFLUX, there is no need to learn the last part of the Equation (1) as the atomic models are trained on the overall atomic energy $E_{\mathrm{IQA}}^{AA^{\prime }}$.

By learning directly from IQA‐partitioned atomic energies and calculating analytical atomic forces from them, FFLUX not only enjoys energy conservation, a trait that is not easy to reach if only forces are predicted [50], but also eliminates the need for training on atomic forces. In contrast, other MLPs often delegate the energy partitioning process to the machine learning algorithm, resulting in site energies without guaranteed connection to a physical reality. Consequently, those MLPs must train using both total energies and atomic forces to get the much‐needed local information about the PES in order to ensure accurate predictions. This reliance arises because the atomic energies derived in these models are mere mathematical constructs, prone to error compensation and lacking physical or chemical interpretability [51]. Moreover, when atomic DFT forces are used as learnable properties, extra care is needed because net zero forces are not guaranteed. Usually, a tighter SCF convergence and a denser grid are required to achieve this. Attaining net‐zero forces is important because data with large net forces often show large atomic force errors [52]. FFLUX's ability to bypass the need for atomic force data enhances the explainability and transferability of its predictions [53], offering a significant edge over traditional MLPs.

### 2.3 Training

FFLUX uses GPR models to predict IQA energies and MMs for each atom. Predictions for an atom depend not only on its position relative to other atoms but also on the environment captured by the atomic model. In other words, surrounding atoms influence each atom's predictions; every atom therefore “knows” its local environment. Because atomic IQA energies include all interaction terms, including exchange and correlation, the IQA energy of an atom can, in principle, account for every interaction provided the models are trained on a sufficiently large system. We demonstrated this in a formamide dimer case study, where moving from monomeric to dimeric models produced substantial improvements [54]. However, the models used in the present paper are monomeric.

To train the models, our in‐house program FEREBUS [55, 56, 57] is used. When the models are ready, predictions [26, 31] are made using equation (2),

(2) $$\begin{array}{l} {\hat{Y}}^{A}=\mu^{A}+\sum_{j=1}^{N_{\text{train}}}a_{j}^{A}\exp\left[-\sum_{h=1}^{N_{\text{feat}}}\theta_{h}^{A}\;r_{d}\left(f_{j,h}^{A},f_{h}^{A}\right)\right]\;\\ r_{d}\left(f_{j,h}^{A},f_{h}^{A}\right)=\left\{\begin{array}{ll} \sin^{2}\;\left(\frac{f_{j,h}^{A}-f_{h}^{A}}{2}\right) & \text{if}\;h>3\;\text{and}\;h\;\mathrm{mod}\;3=0\\ {\left(f_{j,h}^{A}-f_{h}^{A}\right)}^{2} & \text{otherwise} \end{array}\right. \end{array}$$

where after the third feature, the periodic kernel is used to compute feature differences every third feature. For the rest, the RBF‐Cyclic kernel is used. For instance, in methanol, which has 12 features, the following indices are treated by RBF‐Cyclic kernel: 1, 2, 3, 4, 5, 7, 8, 10, 11 and the periodic kernel is used in the case of the feature indices of 6, 9, and 12. The quantity ${\hat{Y}}^{A}$ is the predicted output of property atom *A*, which can be atomic IQA energy or MM. The quantity $\mu^{A}$ is the GP prior mean function. The optimal choice for this function can be found in our previous study [26]. The quantity $a_{j}^{A}$ is the weight of the training point *j*, and $\theta_{h}^{A}$ is the hyperparameter associated with feature *h*, which determines the sensitivity of the model to variations in that feature. The $f_{j,h}^{A}$ is the *h*th feature value of training point *j* and $f_{h}^{A^{\prime }}$ is the $h^{\mathrm{th}}$ feature value of a geometry for which a prediction must be made.

The IQA forces are then calculated using Equation (3),

(3) $$F_{i}^{\Omega }=-\sum_{A}^{N_{\text{atoms}}}\sum_{h=1}^{N_{\text{feat}}}\frac{\partial {\hat{E}}_{\mathrm{IQA}}^{A}}{\partial f_{h}^{A}}\frac{\partial f_{h}^{A}}{\partial \alpha_{i}^{\Omega }}$$

where $F_{i}^{\Omega }$ is the total force acting on a given atom Ω in the direction $i\in \left\{x,y,z\right\}$, arising from the energy contributions and positions (α) of all other atoms.

### 2.4 Overview of Software and Changes

FFLUX is implemented within DL_POLY4, hence the name DL_FFLUX. The FFLUX approach provides flexible MMs, energies, forces, virial, and stress tensor updates to DL_POLY. Subsequently FFLUX relies on DL_POLY to displace atoms while imposing thermostats and barostats on dynamics. The Application Programming Interface (API) of FFLUX is under development, which will allow the use of FFLUX with other MD engines (such as LAMMPS and ASE).

In FFLUX, OpenMP parallelization is employed to optimize key calculations, particularly during the prediction phase, which can often become a bottleneck in simulations. This bottleneck arises because, depending on the system size, thousands of floating‐point operations may be required to generate predictions. Specifically, for each atomic property in a simulation, FFLUX performs a nested loop with dimensions corresponding to the number of training points ($N_{\text{train}}$) and the number of features ($N_{\text{feat}}$), where $N_{\text{feat}}$ is given by 3 N‐6, representing the degrees of freedom in a system with *N* atoms. By utilizing OpenMP parallelization, FFLUX effectively distributes these computations across multiple processors, significantly accelerating the prediction process and improving overall computational efficiency. Moreover, FFLUX uses Message Passing Interface (MPI) [58] parallelization present in DL_POLY 4, where a domain decomposition [59] algorithm is used in the MPI parallelization of DL_POLY. This has been described in detail in previous work [24].

The way MPI/OpenMP hybrid parallelization works in DL_FFLUX is that, given a total number of available CPU cores, the cores are divided into MPI ranks and OpenMP threads. These values are usually chosen such that their product equals the total number of CPU cores used. For example, if 40 cores are available, one could use 8 MPI ranks and 5 OpenMP threads for each MPI rank, or 4 MPI ranks and 10 OpenMP threads.

In practice, during a simulation, the MPI domain decomposition method divides the simulation box into subregions called *domains*. The data and subsequent calculations for the atoms in each domain are assigned to an MPI rank. Within each MPI rank, the assigned work can then be further parallelized using OpenMP threads.

The current paper focuses on targeted refactoring that has made DL_FFLUX's codebase condensed and optimized, yielding fewer source lines and higher efficiency. In the following sections, these changes are explained, and some benchmarks are presented to showcase the speed‐up gained compared to the previous, unoptimized code. Moreover, the benchmarks are extended to a comparison of 4 molecules present in the MD17 database [60].

### 2.5 Changes and Their Necessity

In recent years substantial effort has gone into parallelising DL_FFLUX using MPI and OpenMP [24]. Hence further parallelising the code is not the focus of this paper. However, until recently, the models used in simulations were small, for example, around 100 training points for water [29]. As a result, both the OpenMP strategy and the implementation of prediction and derivative calculations were tuned for models with only a limited number of training points. Under these conditions, certain source‐code issues went largely unnoticed, such as making multiple redundant predictions for each atom–property pair.

The shift toward simulating oligomeric systems and larger drug‐like molecules requires coverage of a much broader region of conformational space, which in turn demands significantly larger training sets. These larger models increase computational cost and slow down simulations, thereby motivating a revision of FFLUX's source code to reduce its overall computational expense. In this work, the Intel VTune Profiler [61] was used. The “hotspots” analysis of VTune interrupts the process at regular 10 ms intervals to record the active instruction pointer and its call stack for each sample. These samples are then aggregated to identify performance‐critical regions of the code. The profiler reports and sorts functions and source lines by CPU time, allowing statistically significant computational bottlenecks (“hotspots”) to be located.

Table 1 shows the hotspots analysis undertaken to identify the most time‐consuming parts of the code. It is evident from this analysis that most of the simulation time is spent in the subroutine *fflux_predict_value* where the ML predictions are calculated. This table includes both user‐defined functions and external runtime or library routines. The [Others] category groups routines without debug symbols, including runtime and external library functions. Because these routines cannot be associated with a single module their module is reported by VTune as N/A. This category includes dynamic loader functions, standard C runtime operations (e.g., memory management and I/O), Intel OpenMP runtime functions, and HPC communication libraries. In some cases, VTune also reports functions as “not available,” which typically results from missing symbol information, compiler inlining, or calls within external libraries. The prediction step became the main computational bottleneck because making GPR predictions requires comparing the features of a given molecular geometry with every geometry in the training set, as seen in Equation (2). Using the kernel function, the model evaluates for each prediction how likely (or similar) the new geometry is relative to each of the stored training geometries. Because realistic models can contain thousands of training geometries, this likelihood‐based comparison must be performed thousands of times at every simulation step thereby making the prediction stage expensive. More precisely, the computational cost grows with the product $N_{\text{atoms}}\times N_{\text{train}}\times N_{\text{feat}}$. Note that $N_{\text{feat}}$ and $N_{\text{atoms}}$ are related by $N_{\text{feat}}=3N_{\text{atoms}}-6$.

**TABLE 1:** Top hotspots when simulating a single paracetamol molecule for 2000 steps (1 fs timestep) with 4000 training points models run in serial, before optimizing the number of calls to *fflux_predict_value* and refactoring the code.

Function	Module	CPU time	% CPU time
fflux_predict_value	DLPOLY.Z	2595.352 s	64.9%
__libm_sin_l9	DLPOLY.Z	763.082 s	19.1%
__libm_sse2_sincos	DLPOLY.Z	436.261 s	10.9%
sign_j	DLPOLY.Z	133.283 s	3.3%
sin	DLPOLY.Z	21.371 s	0.5%
[Others]	N/A^a^	50.922 s	1.3%

^a^ N/A: not available.

A close inspection of the code makes clear that the same atomic prediction was being recomputed inside the pairwise force‐assembly loop in order to obtain the partial derivatives of the predictions with respect to atomic positions, as shown in Equation (3). These repeated evaluations were purely an implementation artifact and were not required by the Gaussian process formulation itself. For example, for paracetamol with 20 atoms, the number of calls to the *fflux_predict_value* routine, required to compute ${\hat{E}}_{\mathrm{IQA}}^{A}$, was $N_{\text{atoms}}^{2}+N_{\text{atoms}}$. The quadratic term follows from the way the derivative calculations were implemented, and the linear term comes from the predictions needed to accumulate the total system IQA energy. This led to many costly, yet redundant, calls to *fflux_predict_value*, especially for larger models. Therefore, we changed the main loop in DL_FFLUX, so that each atom's IQA energy prediction and its feature derivatives are computed only once per MD step, cached, and subsequently reused during the force calculation. This loop also marks the start of the OpenMP parallel region in FFLUX, in which the energy and force calculations for individual atoms are distributed across OpenMP threads. Consequently, the introduction of the cached list required careful consideration to preserve both thread safety and domain safety.

To ensure OpenMP correctness, an explicit FLUSH directive was required after completing the predictions to ensure all OpenMP threads do see the updated IQA‐energy list and the list of their partial derivatives with respect to the features, that is, $\frac{\partial {\hat{E}}_{\mathrm{IQA}}^{A}}{\partial f^{A}}$. This is necessary because Equation (3) shows that the first summation runs over all atoms and their predictions, which is parallelized over multiple threads. Therefore, a thread computing $F_{i}^{\Omega }$ must have access to every prediction within the model.

Predictions for the atoms in this MPI list are also added to the IQA‐energy cache so that threads on different domains can access all required values when calculating forces. Caching predictions instead of recalculating them for every force evaluation removes the quadratic component of the prediction cost. For example, for paracetamol (20 atoms) with a 4000‐point training set, the number of feature‐difference computations required by the likelihood comparison for a single prediction falls dramatically, from 90,720,000 to 4,320,000, that is, a 21‐fold reduction or a factor of $N_{\text{atoms}}+1$ in general. This change yields a substantial improvement in simulation scaling and overall runtime especially for larger models.

According to Table 1 the next major cost was trigonometric evaluations, specifically sin and cos. In our implementation, sin appears in every third feature comparison, while cos appears when computing the partial derivative with respect to a feature (which itself produces a sin term). By using the trigonometric identity $\sin\left(2\theta \right)=2\sin\theta \cos\theta$, we reworked the derivative expressions to avoid an explicit cos evaluation and instead reused the computed sin values, thereby eliminating the costly cosine calls.

The next hotspot was the $\mathrm{sig}n_{j}$ routine (see equation (11) of reference [31]). Because current best practice makes us always set the general power of the feature difference in Equation (2) to two, that is $p_{h}^{A}=2$, this expensive routine is no longer required and was removed. In addition, we reduced the number of conditional statements, especially inside OpenMP regions, which produced further speedups.

For condensed‐phase simulations we applied the same caching logic as that used for IQA energies to the atomic multipole (MM) predictions and their derivatives. Previously, redundant calls to *fflux_predict_value* were made when computing partial derivatives for MMs. We now predict each MM once, store the results, and reuse them whenever needed. This route removes duplicated work and improves performance in condensed phase runs as well, albeit at a higher memory footprint. To further refactor the code, we moved all predictions (IQA and MM) and their feature partial‐derivative calculations into a single OpenMP‐parallelized loop. This reduces the overhead associated with repeatedly forking and synchronizing OpenMP threads.

The final change concerns the calculation of the feature‐difference (fdiff), which is used for prediction as can be seen in Equation (2). Because the datasets used to train IQA and MM models are typically identical, there is no need to recompute fdiff separately for every atomic property. We therefore modified the code such that the fdiff list is computed once per atom and reused for subsequent predictions provided all properties share the same training set. This way of working is particularly beneficial in condensed‐phase runs: for simulations with *L*′ = 0, 1, 2, 3 or 4, the number of extra predictions required to obtain atomic MMs is 1, 4, 9, 16 and 25, respectively. Here, *L*′ = 0 corresponds to charge–charge interactions, *L*′ = 1 to charge–charge, charge–dipole and dipole–dipole, and *L*′ = 4 contains interactions up to hexadecapole‐hexadecapole interactions.

In addition to performance optimizations, we refactored the code to follow clean code principles. For instance, each function now has a single, well‐defined responsibility. Figure S1 of the Supporting Information (SI) provides a detailed flowchart of the refactored DL_FFLUX.

## 3 Results and Discussion

We first report the speed‐up achieved by the optimized version relative to the previous, unoptimized implementation across several systems. Finally, we compare our performance with other MLPs on four molecules from the MD17 dataset.

The level of theory used for all the models is B3LYP/aug‐cc‐pVTZ, where GAUSSIAN16 [62] was used to generate wavefunction files, and AIMAll (version 19.10.12) to perform QTAIM and the IQA energy partitioning. Timings were measured on a full node, a dual‐socket Intel Xeon Gold 6230 node (40 cores in total) running at 2.10 GHz. For each case we ran five trials and reported the fastest observed time, that is, that with the least time perturbation due to system processes.

To measure the speed‐up from the optimizations, we performed simulations of isolated molecules in vacuum and condensed‐phase NVT simulations (time step 0.5 fs, Nosé–Hoover thermostat [63]). The molecules are ammonia (4 atoms), methanol (6 atoms), ethanol (9 atoms), paracetamol (20 atoms), nicotine (26 atoms), and tetra‐alanine (TALA) (42 atoms). Here, we varied the number of training points and the number of OpenMP threads for the reported timings.

**FIGURE 1:**
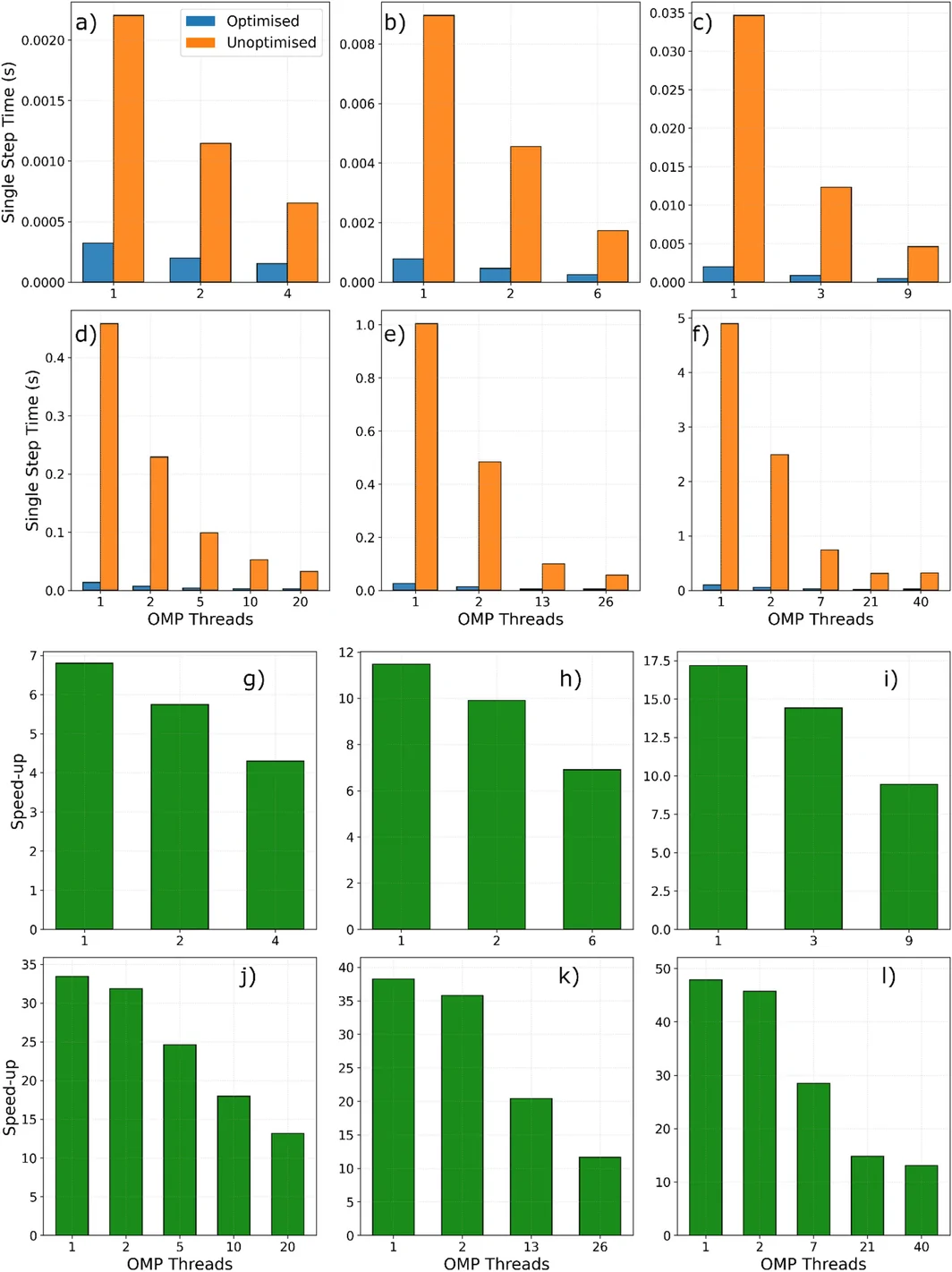
Benchmarking of 6 molecules, each using a 1000‐point training model, for the optimized and unoptimized version of DL_FFLUX. Panels (a)–(f) respectively show the results for systems ammonia, methanol, ethanol, paracetamol, nicotine and TALA. Panels (a)–(f) compare the wall‐clock time per MD step for the optimized (blue) and unoptimized (orange) versions of DL_FFLUX using different OpenMP threads. Panels (g)–(l) present the corresponding speed‐up, defined as (unoptimized time)/(optimized time). Their panel order matches that of panels (a)–(f). Figure S2 of the SI shows the same benchmark using the largest available models: all systems use 8000 training points except TALA with 6000 training points. The single‐thread speed‐ups in panels (g)–(l) are 9.3, 14.4, 21.7, 51.6, 66.7 and 93.2, respectively. These values show a more pronounced improvement than for the 1000‐point models, indicating that the optimized version scales much better with model size than the unoptimized version. Figure S3 presents the complete serial speed‐up benchmark for all systems across model sizes ranging from 1000 to 8000 training points. The left heatmap shows simulation frames per second (FPS) for the unoptimized (orange) and optimized (blue) versions. The right heatmap shows the speed‐up obtained in each case. Overall, the numbers show that every system achieves a substantial speed‐up. Improvements are most pronounced for models with more training points, and for molecules with a larger number of atoms. This overall result indicates that the optimizations benefit larger systems the most, which is pleasing news.

The upper part of Figure 1 (panels (a)–(f)) shows the wall‐clock time per MD step for the six systems using the optimized and unoptimized versions of DL_FFLUX as a function of the number of OpenMP threads, with all models using 1000 training points. For the same 6 systems the lower part shows the speed‐up, defined as $\text{peedup}=\frac{\mathrm{tim}e_{\text{unoptimised}}}{\mathrm{tim}e_{\text{optimised}}}$. For single‐thread (serial) runs, the speed‐ups shown in panels (g)–(l) are 6.8, 11.5, 17.2, 33.4, 38.3 and 49.1, respectively and more generally, as the system size (number of atoms) increases, the computational cost rises. However, the optimized code reduces this cost substantially. Note that the measured speed‐up tends to fall as the number of OpenMP threads increases. This is because the optimizations removed many redundant computations, leaving less parallel work to distribute, with fewer heavy tasks per thread.

To better understand this reduction in parallel speed‐up, we need to remember Amdahl's law, which states that, under ideal parallelization (no overhead), the maximum speed‐up, denoted *S*, that is achievable with *N* cores is

(4) $$S\left(N\right)=\frac{1}{\left(1-p\right)+\frac{p}{N}}$$

where *p* is the fraction of the workload that is perfectly parallelisable and 1−p is the strictly serial fraction. In the limit N→∞, the speed‐up becomes

(5) $$S_{\max}=\frac{1}{\left(1-p\right)}$$

showing that the serial portion of the code ultimately bounds the achievable speed‐up. Note that real implementations also incur overheads, which further reduce attainable gains. As stated earlier, the aim of this work is not to push parallelization further but to refactor and optimize the code to reduce overall cost.

The next set of tests consists of condensed‐phase simulations: pure water (512 molecules, models with 100 training points) and pure ethanol (64 molecules, models with 3000 training points). For these runs, we varied the number of OpenMP threads and MPI ranks, and we considered the multipole moment interaction rank *L*′ to be 0, 1, or 2.

**FIGURE 2:**
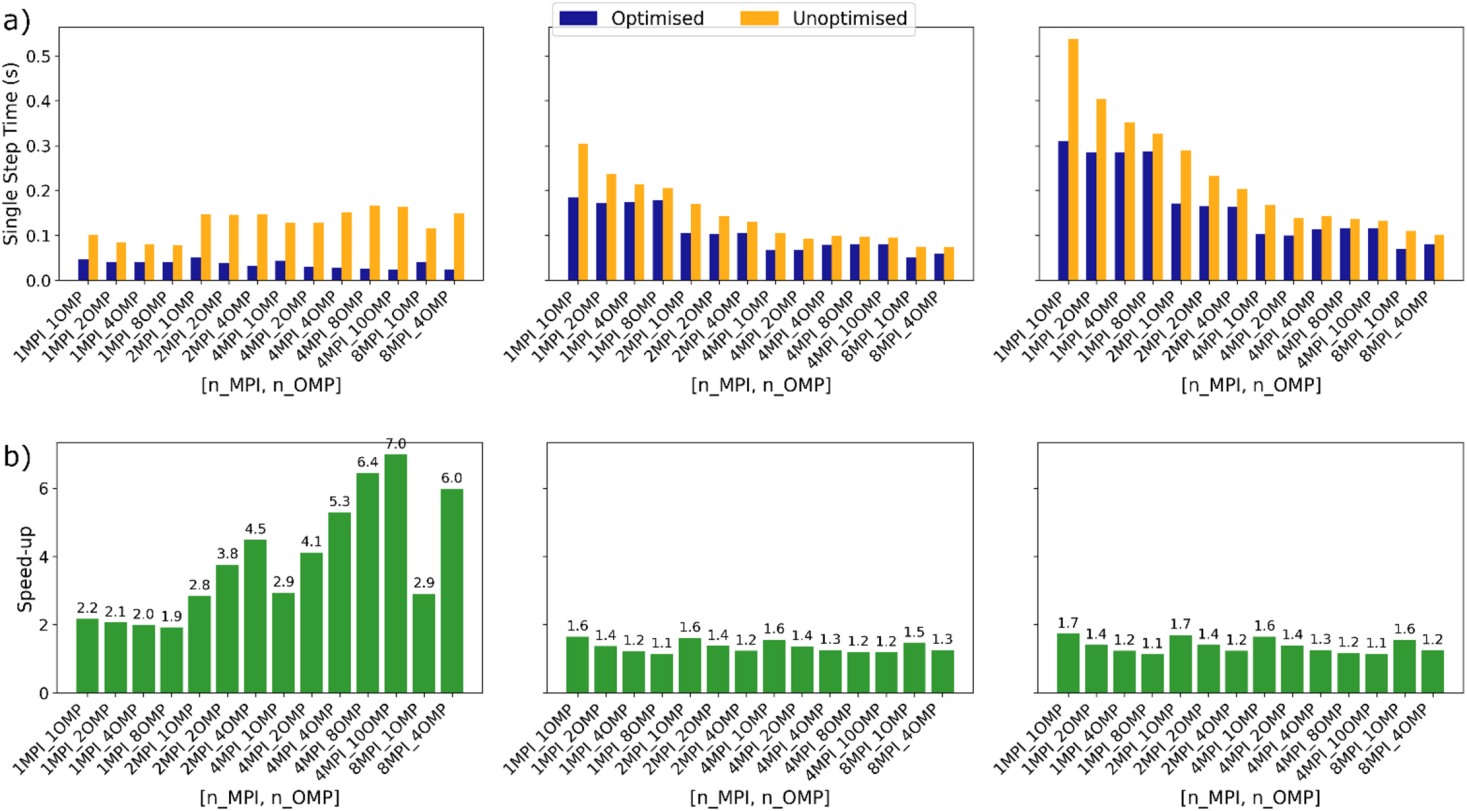
Benchmarking results of 512 water molecules with 100‐points models using the optimized and unoptimized versions of DL_FFLUX: (left plots) *L*′ = 0, (middle) *L*′ = 1 and (right) *L*′ = 2. Panel (a) compares the wall‐clock time per MD step for the optimized (blue) and unoptimized (orange) versions of DL_FFLUX using different *L*′ values, MPI ranks and OpenMP (OMP) threads. Panel (b) presents the corresponding speed‐up, defined as the ratio (unoptimized time)/(optimized time).

Figure 2 shows how the optimized and unoptimized DL_FFLUX versions perform on 512 water molecules. Let us remember that multiplying the number of MPI ranks by the number of OpenMP threads returns a certain number of CPU cores. The upper plots (a) demonstrate that, for a given number of CPU cores, using more MPI ranks than OpenMP threads results in a larger speed‐up. This conclusion can be reached by several observations, most easily made in the (b) panels, which show the speed‐ups as ratios of (unoptimized time)/(optimized time). For example, comparing a 1 MPI_8 OpenMP run with an 8 MPI_1 OpenMP one shows that the latter renders a larger speed‐up than the former, that is, 2.9 versus 1.9 respectively for *L*′ = 0. The (b) panels show that the optimized DL_FFLUX is faster for all combinations of MPI ranks, OpenMP threads and *L*′ values. However, the performance gap narrows for *L*′ values of 1 and 2. The largest speed‐up ever reached for the new versus the old implementation is 7, as seen for 4 MPI ranks and 10 OpenMP threads when *L*′ = 0.

**FIGURE 3:**
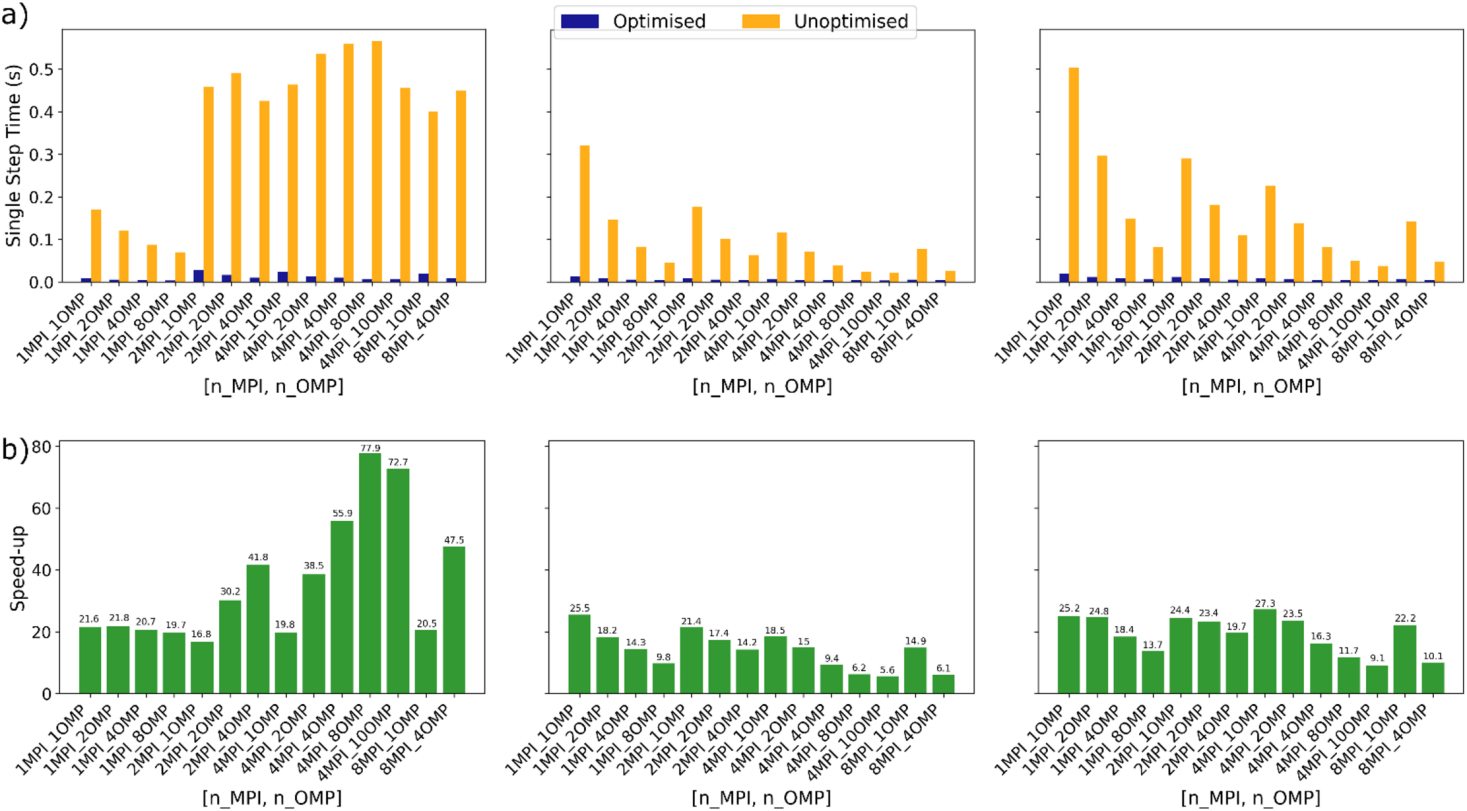
Benchmarking speed‐ups for 64 ethanol molecules with 3000‐points models using the optimized and unoptimized versions of DL_FFLUX: (left plots) *L*′ = 0, (middle) *L*′ = 1, and (right) *L*′ = 2. Panel (a) compares the wall‐clock time per MD step for the optimized (blue) and unoptimized (orange) versions of DL_FFLUX using different *L*′ values, MPI ranks and OpenMP (OMP) threads. Panel (b) presents the corresponding speed‐up, defined as the ratio (unoptimized time)/(optimized time).

Figure 3 follows the same procedure as Figure 2 but simulates 64 ethanol molecules using 3000‐point models. The 3 top plots marked (a) show that simulation time scales better when using more OpenMP threads than MPI ranks. This conclusion is opposite to that for the water results in Figure 2, where using more MPI ranks gave better speed‐up. The reason is that water models are small (100 training points) while FFLUX's OpenMP parallelization is implemented within the prediction loop, whose computational cost depends directly on the number of training points. For such small models, this prediction step accounts for a relatively small fraction of the total execution time, limiting the benefit of OpenMP parallelization. In contrast, MPI parallelises larger parts of the code and therefore reduces the overall runtime more effectively. However, in the ethanol simulation the models are larger and contain more atoms (and thus more features), making the prediction step substantially more expensive. As a result, OpenMP parallelization becomes more effective during the prediction step, thereby reducing the overall computational cost.

### 3.1 Benchmarking on the MD17 Dataset

Next, we benchmarked four molecules from the MD17 dataset, namely ethanol, naphthalene, salicylic acid and aspirin, using the optimized version of DL_FFLUX. The MD17 dataset was generated by AIMD simulations at 500 K for several small and medium‐sized molecules. However, we did not use MD17 data for training, nor for the internal validation set, when building our models. At high temperature, (AI)MD does not sample rare events or transitions between stable conformers well, as shown for salicylic acid in Figure S4. In particular, the MD17 trajectory fails to sample conformers in which the intramolecular hydrogen bond is absent, or the carboxylic acid group is rotated. Consequently, a model trained solely on MD17 data would not be exposed to these physically relevant regions of conformational space, thereby limiting the assessment of its robustness. In order to improve conformational coverage and avoid holes in the PES, we ran metadynamics and selected the most diverse subset of sampled geometries to build 2000‐point models. Another reason for excluding MD17 from training is that the dataset shows autocorrelation in the time series. It is recommended to use no more than 1000 training points from MD17 when building models [64, 65].

Table 2 compares FPS (i.e., speed) and stability of simulations performed with different MLPs for 4 molecules in the MD17 dataset. To calculate the stability and FPS for each molecule, NVT MD simulations were performed at 500 K with a Nosé–Hoover thermostat and a time step of 0.5 fs for 600,000 steps (300 ps). Stability is assessed by comparing bond lengths in the encountered geometries with those in the optimized reference geometry. This comparison is performed for every bonded atom pair. Further details are provided in Section 5 of the SI. In this benchmark, DL_FFLUX uses the maximum number of OpenMP threads ($N_{\text{atom}}$) for each molecule. We observe that for every molecule, DL_FFLUX's FPS is an order of magnitude larger than all other implementations. This is particularly true for ethanol, where DL_FFLUX is 9.6 times faster than MD‐ET, which is the fastest after DL_FFLUX.

**TABLE 2:** (a) Stability and (b) frame per second (FPS) for different MLPs on 4 MD17 molecules.

	DP‐SE [66]	SchNet [12]	DimeNet [67]	PaiNN [68]	SphereNet [69]	ForceNet [70]	GemNet‐dT [71]	NequIP [13]	MD‐ET [72]	DL_FFLUX
(a) Stability										
Aspirin	9	26	54	159	141	182	192	300	300	300
Ethanol	300	247	26	86	33	300	300	300	300	300
Naphthalene	246	18	85	300	6	300	25	300	300	300
Salicylic acid	300	300	73	281	36	1	94	300	300	300
Average	213.8	147.8	60.0	206.5	54	195.8	152.8	300	300	300
(b) FPS (frames per second)										
Aspirin	88.0	108.9	20.6	85.8	17.5	137.3	56.8	8.4	159.8	288.1
Ethanol	101.0	112.6	21.4	87.3	30.5	141.1	54.3	8.9	148.1	1420.0
Naphthalene	109.3	110.9	19.1	92.8	18.3	140.2	53.5	8.2	150.2	418.4
Salicylic acid	94.6	111.7	19.4	90.5	21.4	143.2	52.4	8.4	147.3	530.0
Average	98.2	111.0	20.1	89.1	21.9	140.5	54.3	8.5	151.4	664.1

*Note:* FPS is measured as the number of simulation steps per second. Higher values indicate better performance for both metrics. Details of the stability assessment are provided in Section 5 of the SI.

It is also useful to assess model accuracy, as shown in Table 3, which benchmarks DL_FFLUX and other MLP models on a test set of 10,000 points obtaining their force mean absolute error (MAE) (in meV/Å). The higher errors reported for DL_FFLUX can be attributed to the fact that we did not train on MD17, so testing on MD17 increases the MAE of the forces. In addition, DL_FFLUX models were trained only on energies. The forces were not included during training. Forces are obtained by differentiating the predicted energies (see Equation (3)), which typically yields larger MAEs for forces than for energies. Incorporating force information as derivative observations during Gaussian process training has been demonstrated previously for FFLUX, where energy and derivative observations were incorporated within a unified Gaussian process model [73]. We are currently investigating an approach to FFLUX where force information will be incorporated into the loss function in the training process while retaining atomic energies as the primary model outputs and obtaining forces through the existing energy‐gradient chain rule relationship. This work will assess whether force‐informed training improves the accuracy of the predicted atomic energies and the resulting forces, while also evaluating its impact on training cost.

**TABLE 3:** Force MAE (meV/Å) on 10,000 MD17 geometries.

	DP‐SE	SchNet	DimeNet	PaiNN	SphereNet	ForceNet	GemNet‐dT	NequIP	MD‐ET	DL_FFLUX	DL_FFLUX^a^
Aspirin	21.0	35.6	10.0	9.2	3.4	22.1	5.1	2.3	4.2	622.0	5.6
Ethanol	8.9	16.8	4.2	5.0	1.7	14.9	1.7	1.3	1.0	223.6	0.1
Naphthalene	13.4	22.5	5.7	3.8	1.5	9.9	1.9	1.1	2.3	186.4	1.0
Salicylic acid	14.9	26.3	9.6	6.5	2.6	12.8	4.0	1.6	2.8	441.3	1.7
Average	14.6	25.3	7.4	6.1	2.3	15.0	3.2	1.6	2.6	368.3	2.1

^a^ DL_FFLUX energy MAE (kJ/mol).

Finally, we note that the column on the utmost right shows that the molecular energy MAE is below chemical accuracy (1 kcal/mol) for all systems except aspirin, where the error is slightly above this accuracy at 5.6 kJ/mol. We are actively working to improve FFLUX force accuracy. As we have proved before, FFLUX produces correct forces that exactly reproduce the known stationary points of the alanine dipeptide [26].

## 4 Conclusions

Having an accurate and fast potential that can be used to simulate and study the dynamics of molecules is most sought after. Unfortunately, most MLPs neglect speed and do not optimize their code, which can lead to slow simulations that use much computation and energy. That lack of enthusiasm stems from the fast‐moving nature of the machine learning community and the constant need to add new features to the code. These additions often require major changes to the source, so spending effort to optimize a single code snapshot usually does not make sense. Here, we challenged this assumption and showed that by refactoring and optimizing the source code, the simulations carried out with DL_FFLUX have become 6.8–49 times faster. By comparison with other MLPs on 4 molecules selected from the MD17 dataset, we have shown DL_FFLUX to be the fastest among the tested MLPs whilst its predictions remain accurate and simulations stable. The changes to the source code were mainly driven by hotspots identified with the VTune Intel profiler, with a focus on optimizing and refactoring the code.

## Funding

This work was supported by UK Research and Innovation (EP/X024393/1).

## Supporting information


**Figure S1:** Refactored DL_FFLUX: process flow and module responsibilities. DL_POLY subroutines (blue) perform the classical MD tasks while FFLUX subroutines (red) handle machine‐learning predictions and flexible MM‐Ewald electrostatics.
**Figure S2:** Benchmarking of 6 molecules, each using an 8000‐point training model, for the optimized and unoptimized versions of DL_FFLUX. Panels (a)–(f) respectively show the systems ammonia, methanol, ethanol, paracetamol, nicotine and tetra‐alanine (TALA). Panels (a)–(f) compare the wall‐clock time per MD step for the optimized (blue) and unoptimized (orange) versions of DL_FFLUX using different OMP threads. Panels (g)–(l) present the corresponding speed‐up, defined as the ratio (unoptimized time)/(optimized time). Their panel order matches that of panels (a)–(f).
**Figure S3:** Serial benchmarking of 6 molecules using different versions of DL_FFLUX. The systems are ammonia, methanol, ethanol, paracetamol, nicotine and TALA, respectively, with different model sizes ranging from 1000 to 8000. Panel (a) compares the heatmap frame per second (FPS) for the optimized (blue) and unoptimized (orange) versions of DL_FFLUX on a single thread. Panel (b) presents the corresponding speed‐up, defined as the ratio (unoptimized time)/(optimized time).
**Figure S4:** Mist plots containing 10,000 points for four molecules (a) and (b) are ethanol, (c) and (d) are naphthalene, (e) and (f) are salicylic acid, and (g) and (h) are aspirin. The left‐column plots are random samples from the MD17 dataset and were used as the test set. The right‐column plots come from WTMetaD at 300 K, except panel (d) (naphthalene), which comes from an unbiased simulation at 500 K.

## Data Availability

The data underlying this study are available from the Mendeley data repository (https://data.mendeley.com/datasets/46pcdd5gdv/1). The dataset contains 10,000 geometries sampled using metadynamics, along with atomic IQA energies, atomic charges, and molecular mechanics forces calculated at the B3LYP/aug‐cc‐pVTZ level of theory. The trained models used in this work are also provided. The FEREBUS and DL_FFLUX programs are accessible at https://github.com/popelier‐group/FEREBUS_CPU and https://github.com/popeliergroup/DL_FFLUX‐v3, respectively. DL_FFLUX code can also be cited using the following https://doi.org/10.5281/zenodo.18713944.
