# Supplementary material for: DL_FFLUX Refactored: Accelerating Quantum Chemical Topology Simulations With Optimized Parallelization
Source: J Comput Chem. 2026 Sep 8;47(24):e70501. doi: 10.1002/jcc.70501 (PMC13552095; doi:10.1002/jcc.70501)
Supplement: Supplementary file 1 [file JCC-47-0-s001.docx]

Supporting Information

**DL_FFLUX Refactored: Accelerating Quantum Chemical Topology Simulations with Optimised Parallelisation**

Mohamadhosein Nosratjoo^†^, Michael K. Bane^‡,¶^ and Paul L.A. Popelier^*,†^

† Department of Chemistry, The University of Manchester,

Manchester, M13 9PL, Great Britain

‡ High End Compute LTD, Manchester M20 5PD, Great Britain

¶Department of Computing and Mathematics, Manchester Metropolitan University, Manchester M15 6BH, Great Britain

E-mail: paul.popelier@manchester.ac.uk

**1. Details on DL_FFLUX**

DL_FFLUX is a molecular dynamics package, written in Fortran90, which contains the machine learning potential (MLP) FFLUX. The program DL_FFLUX is composed of FFLUX and DL_POLY4, where about 8 % of the subroutines belong to FFLUX, while the remaining 344 subroutines are part of DL_POLY4.

The code has become substantially more modular after our refactoring (see Fig.S1). The central idea of FFLUX is to predict atomic properties, energies and multipole moments (MMs), via Gaussian Process regression (GPR) models for each atomic property. This is possible by learning the atomic energies and MMs obtained from partitioning real space via the quantum theory of atoms in molecules (QTAIM) and the Interacting Quantum Atoms (IQA) method, which partitions the total energy into atomic contributions. This per-atom approach gives FFLUX fine-grained control over simulations and makes it easier to detect and diagnose model failures. As an example, FFLUX successfully recovered the physical structure at the first couple of picoseconds in simulations starting from never-trained, unphysical geometries, as shown in reference ^1^.

Figure S1 illustrates how FFLUX subroutines interact with DL_POLY. The interface has two stages. The first is initialisation, run once per simulation: it reads instructions and keywords and loads the model data (weights, hyperparameters, mean values, training features, etc.). The second stage runs every molecular dynamics (MD) step: features are calculated, IQA energies and multipole moments are predicted, and the corresponding forces, stress and virial are computed. We have implemented this approach in an API that interfaces with the Atomic Simulation Environment (ASE) ^2^ and demonstrated it by performing geometry optimisation of the alanine dipeptide, successfully locating the target minimum^1^.

**Figure S1.** Refactored DL_FFLUX: process flow and module responsibilities. DL_POLY subroutines (blue) perform the classical MD tasks while FFLUX subroutines (red) handle machine-learning predictions and flexible MM-Ewald electrostatics.

**2. Additional data on benchmarks**

The earlier, unoptimised DL_FFLUX code worked well enough for small molecules (e.g. water, formamide) but it became very costly for larger systems. Our refactoring and optimisation target this problem such that we can run amino acids and oligopeptides more efficiently. Larger systems introduce more atoms, and their models require many more training points to capture the systems’ complex potential energy surfaces. Both the system size and the model size raise the per-atom prediction time in FFLUX and thus increase the total simulation time in DL_FFLUX.

Figure S2 clearly shows that, after applying the changes explained in the main paper, the simulations are between 7.5 and 93.2 times faster, where the speed-up is more pronounced for larger systems. Furthermore, comparing Figure 1 (1,000 points) in the main paper and Figure S2 (8,000 points) shows that models with a higher number of training points benefit more from the changes applied to the code.

**Figure S2**. Benchmarking of 6 molecules, each using an 8,000-point training model, for the optimised and unoptimised versions of DL_FFLUX. Panels (a)–(f) respectively show the systems ammonia, methanol, ethanol, paracetamol, nicotine and tetra-alanine (TALA). Panels (a)–(f) compare the wall-clock time per MD step for the optimised (blue) and unoptimised (orange) versions of DL_FFLUX using different OMP threads. Panels (g)–(l) present the corresponding speed-up, defined as the ratio (unoptimised time)/(optimised time). Their panel order matches that of panels (a)–(f).

Panel (a) of Figure S3 compares frames per second (FPS) for optimised and unoptimised single-molecule serial simulations across several systems and model sizes (1,000–8,000 points). Panel (b) shows the corresponding speed-ups. The results clearly show that larger systems and larger models improve most after optimisation.

**Figure S3.** Serial benchmarking of 6 molecules using different versions of DL_FFLUX. The systems are ammonia, methanol, ethanol, paracetamol, nicotine and TALA, respectively, with different model sizes ranging from 1,000 to 8,000. Panel (a) compares the heatmap frame per second (FPS) for the optimised (blue) and unoptimised (orange) versions of DL_FFLUX on a single thread. Panel (b) presents the corresponding speed-up, defined as the ratio (unoptimised time)/(optimised time).

**3. Sampling information**

In this study, models are trained on geometries sampled from well-tempered metadynamics (WTMetaD) ^3^. By introducing a history-dependent bias potential, WTMetaD discourages the simulation from revisiting the visited geometries, and makes the sampling of rare events more accessible compared to unbiased MD simulations. The simulations were performed at 300 K in the canonical NVT ensemble at the GFN2-xTB ^4^ level with the Langevin thermostat, which introduces a random force, promoting a better sampling of geometries with a friction coefficient of 0.01 $fs^{-1}$. Each simulation was run for 10 ns with a timestep of 1 fs, where the geometries were sampled every 1 ps. The collective variables chosen for WTMetaD were calculated in the PLUMED ^5^ interface with ASE were dihedral angles since they are the slow-varying motions for each molecule. Next, we generate subsamples from this data by using a diversity-aware sub-sampler (DAS) ^1^ code, which sorts the data by comparing their RMSD with each other. These steps are crucial to shield the models from extrapolations. Figure 4 demonstrates the mist plots of 10,000 geometries randomly sampled from the MD17 dataset on the left (a, c, e, g) and the same number of points sampled from the WTMetaD (except naphthalene) on the right (b, d, f, h). In order to generate these plots, all the structures are superimposed after applying the Kabsch-Umeyama algorithm ^6^. The systems shown are ethanol, naphthalene, salicylic acid, and aspirin.

Naphthalene was treated differently. Its simulation was performed at 500 K in the canonical NVT ensemble at the GFN2-xTB level with the Langevin thermostat. The naphthalene run lasted 2 ns, and geometries were sampled every 100 fs.

The failure of MD17 to capture all relevant conformers is quite visible in the case of salicylic acid, where MD17 (e) completely misses the conformers without intramolecular hydrogen bonds and those where the carboxylic acid functional group is rotated. This means that the stability (robustness) test done on models that are only trained on the MD17 dataset is meaningless, since even if full stability is achieved, it was because the simulation time was not enough for a transition to happen and cause the model to extrapolate and inevitably break.

**Figure S4**. Mist plots containing 10,000 points for four molecules (a) and (b) are ethanol, (c) and (d) are naphthalene, (e) and (f) are salicylic acid, and (g) and (h) are aspirin. The left-column plots are random samples from the MD17 dataset and were used as the test set. The right-column plots come from WTMetaD at 300 K, except panel (d) (naphthalene), which comes from an unbiased simulation at 500 K.

**5. Stability calculation**

Stability, or robustness, is the ability of a model to produce and maintain physically reasonable geometries during a simulation. This robustness depends strongly on how well the underlying potential energy surface (PES) is sampled. If important regions of the PES are unsampled or undersampled then the model must extrapolate there and can provide wrong forces that lead to unphysical geometries. Poor learning of the PES can also cause incorrect force predictions and unstable behaviour. Finally, a low RMSE or MAE does not guarantee stability. Indeed, a model can score well on these error metrics yet still fail if it has not covered the relevant parts of the PES or cannot recover when driven into extrapolative regions.

In assess the stability of simulations done using MLPs, usually a geometry at time step *T* is considered unphysical if ^7,8^

where *B* is the set of all bonds, $\mathbf{x}_{i}$ the position vector of atom *i*, and ||…|| denotes the Euclidean norm. For every bonded atom pair (i,j), the bond length $\|\mathbf{x}_{i}(T)-\mathbf{x}_{j}(T)\|$ at time step *T* is compared with the corresponding bond length ($b_{i,j}$) in the optimised geometry. The absolute difference is computed for every bond, and the maximum deviation over all bonded atom pairs is used to assess the stability of the geometry. If this maximum deviation exceeds a threshold ($\Delta$) then the geometry is classified as unphysical. The value of $\Delta$ is usually set to 0.5 Å. However, we refined this comparison such that the threshold depends on the type of bond being considered

with $\gamma=1.65$. The top equation decides if an explosion has happened while the bottom equation decides on implosion.

**References**

1. B. K. Isamura, O. Aten, M. Nosratjoo, P. L. A. Popelier, Commun. Chem. **2026**, 9, 138.

2. A. Hjorth Larsen, J. Jørgen Mortensen, J. Blomqvist, I. E. Castelli, R. Christensen, M. Dułak, J. Friis, M. N. Groves, B. Hammer, C. Hargus, J. Phys. Condens. Matter **2017**, 29, 273002.

3. A. Barducci, G. Bussi, M. Parrinello, Phys. Rev. Lett. **2008**, 100, 020603.

4. C. Bannwarth, S. Ehlert, S. Grimme, J. Chem. Theory Comput. **2019**, 15, 1652-1671.

5. M. Bonomi, D. Branduardi, G. Bussi, C. Camilloni, D. Provasi, P. Raiteri, D. Donadio, F. Marinelli, F. Pietrucci, R. A. Broglia, Comput. Phys. Commun. **2009**, 180, 1961-1972.

6. S. Umeyama, *IEEE Trans. Pattern Anal. Mach. Intell.* **1991**, 13, 376–380.

7.C. D. Williams, J. Kalayan, N. A. Burton, R. A. Bryce, Chem. Sci. **2024**, 15, 12780-12795.

8. X. Fu, Z. Wu, W. Wang, T. Xie, S. Keten, R. Gómez-Bombarelli, T. Jaakkola, arXiv **2022**, arXiv:2210.07237. <https://arxiv.org/abs/2210.07237>.
